# Ticks of the Genus *Amblyomma* and Lice of the Genus *Eutrichophilus*: Potential Vectors of Brazilian Porcupinepox Virus

**DOI:** 10.3390/pathogens14080809

**Published:** 2025-08-15

**Authors:** Nathana Beatriz Martins, Matias Pablo Juan Szabó, Julio Cesar de Souza Junior, Mario Henrique Alves, Marcio de Barros Bandarra, Paulo Eduardo Brandão, Aline Santana da Hora

**Affiliations:** 1 Veterinary Etiological Investigation Laboratory, School of Veterinary Medicine and Animal Health, Federal University of Uberlandia, Uberlandia 38405-302, MG, Brazil; nathanamartins@ufu.br; 2 Ixodology Laboratory, School of Veterinary Medicine and Animal Health, Federal University of Uberlandia, Uberlandia 38405-302, MG, Brazil; szabo@ufu.br; 3 Department of Veterinary Medicine, Regional University of Blumenau, Blumenau 89030-903, SC, Brazil; juliosouza@furb.br; 4 Department of Veterinary Medicine, University of Bari, 70121 Bari, Italy; mariohalves@live.com; 5 Wild Animal Laboratory, School of Veterinary Medicine and Animal Health, Federal University of Uberlandia, Uberlandia 38405-314, MG, Brazil; bandarramb@ufu.br; 6 Department of Preventive Veterinary Medicine and Animal Health, School of Veterinary Medicine, University of São Paulo, São Paulo 05339-003, SP, Brazil; paulo7926@usp.br

**Keywords:** emerging viral disease, Erethizontidae, viral transmission vector, poxvirus

## Abstract

Brazilian porcupinepox virus (BPoPV) is a recently described pathogen associated with severe cutaneous and systemic disease in *Coendou* spp. porcupines, posing potential conservation and zoonotic risks. Given the solitary behavior of porcupines and the unclear mechanisms of BPoPV transmission, this study investigated the presence of BPoPV DNA in porcupines and their associated ectoparasites (ticks and lice). We analyzed ticks and lice collected from 17 porcupines (*C. longicaudatus* and *C. spinosus*), with or without clinical signs of BPoPV infection. Ectoparasites were identified morphologically, separated into distinct pools for ticks and lice by host, and screened by PCR. BPoPV DNA was detected in all symptomatic porcupines and their ectoparasites—including *Amblyomma longirostre*, *A. sculptum* ticks, and *Eutrichophilus* spp. lice—except for one tick pool. Notably, an asymptomatic, BPoPV-negative porcupine harbored *A. longirostre* ticks that tested positive for the virus. Sequencing confirmed 100% identity with the BPoPV reference strain. These findings suggest that *Eutrichophilus* lice, *A. sculptum*, and particularly *A. longirostre* ticks may play a role in BPoPV transmission. Further studies are needed to elucidate whether these ectoparasites act as biological or mechanical vectors and to assess the zoonotic potential of BPoPV in contact with humans and domestic animals.

## 1. Introduction

Rodents represent the most diverse order of living mammals, comprising over 40% of all mammalian species [1]. Notably, they are among the taxa most frequently implicated in zoonotic disease transmission, with associations to approximately 85 unique zoonotic pathogens [2]. Brazil harbors a particularly high diversity of wild Neotropical porcupines (family Erethizontidae, order Rodentia), with 12 recognized species [3]. Recent phylogenetic studies have redefined the taxonomy of the *Coendou prehensilis* complex, restricting *C. prehensilis* to the northern Atlantic Forest and identifying two newly described species: *C. baturitensis*, found in the eastern Amazon and montane forest enclaves within the Caatinga biome, and *C. longicaudatus*, distributed across the Amazon, Cerrado, and Chaco regions [4]. Some of these species are now at risk of extinction, highlighting the urgency of studying their ecology and associated pathogens.

In certain regions of Brazil, porcupines are occasionally hunted for human consumption [5], raising concerns about potential zoonotic spillover of infectious pathogens. Recently, a novel and highly pathogenic poxvirus—designated Brazilian porcupinepox virus (BPoPV)—was identified in the *Coendou prehensilis* complex (specifically *C. longicaudatus*), causing severe cutaneous and systemic disease with high fatality rates [6]. To date, BPoPV infections have been reported in porcupines across three Brazilian states [6,7,8], highlighting its dual role as a potential emerging zoonotic agent and a conservation threat to Neotropical porcupine populations.

Ticks are well-recognized vectors of diverse pathogens, including protozoa, rickettsiae, spirochaetes, and viruses of significant public and veterinary health concern. Among tick-borne viral diseases, several agents stand out for their clinical importance: tick-borne encephalitis virus, Crimean-Congo hemorrhagic fever virus, and Kyasanur Forest disease virus in humans; African swine fever virus and Nairobi sheep disease virus in animals [9]; along with emerging threats such as Powassan virus, deer tick virus, Bourbon virus, Heartland virus, and severe fever with thrombocytopenia syndrome virus (SFTSV) [10,11,12,13,14]. Similarly, lice serve as important vectors in disease transmission, associated with human pathogens including *Bartonella quintana*, *Borrelia recurrentis*, and *Rickettsia prowazekii* [15]. In veterinary contexts, the pig louse (*Haematopinus suis*) has been implicated as a mechanical vector for swinepox virus (SWPV), which causes proliferative dermatitis in swine [16,17,18]. This established role of ectoparasites in pathogen transmission underscores the importance of investigating their potential involvement in BPoPV spread.

Poxviruses are notable for their broad host range and diverse transmission modes, including arthropod-borne spread. Compelling evidence demonstrates that ticks can serve as mechanical, transstadial, or even transovarial vectors for poxviruses, as exemplified by lumpy skin disease virus (LSDV)—a chordopoxvirus phylogenetically related to BPoPV [19,20,21]. Similarly, lice and stable flies have been implicated in swinepox virus (SWPV) outbreaks among domestic pigs and wild boars, particularly in settings with severe ectoparasite infestations and poor sanitary conditions [16,17,18]. The vector competence of ticks for poxviruses may be enhanced by several factors: (1) prolonged epithelial contact during feeding, providing direct access to viral target cells; and (2) immunomodulatory effects of tick saliva that potentially facilitate viral establishment [22]. These documented mechanisms in related poxvirus-ectoparasite systems strongly support the hypothesis that *Amblyomma* ticks and *Eutrichophilus* lice could mediate BPoPV transmission among porcupines, a scenario particularly plausible given the host’s solitary behavior, which limits direct conspecific contact.

Due to the solitary nature of wild porcupines [23], the transmission mechanisms of BPoPV between conspecifics remain poorly understood. We hypothesized that ectoparasites—particularly ticks and lice—might serve as mechanical vectors facilitating viral spread. To test this hypothesis, we conducted molecular screening for BPoPV in ticks and lice collected from both clinically affected and asymptomatic individuals of two porcupine species: *Coendou longicaudatus* and *C. spinosus*.

## 2. Materials and Methods

### 2.1. Samples

A total of 17 porcupines, including *C. longicaudatus* (*n* = 16) and *C. spinosus* (*n* = 1), were sampled. All samples in this study were obtained from the Veterinary Etiological Investigation Laboratory (LIvE Vet) biobank, located in Uberlândia, Minas Gerais State, Brazil. The samples were collected from porcupines admitted to referral rescue centers due to illness or the need for clinical evaluation prior to reintroduction into the wild. No animals were captured or handled specifically for the purposes of this study.

Cutaneous clinical signs characterized by multifocal skin thickening, erythema, and edema with predilection for distal limbs and mucocutaneous junctions (periocular regions, muzzle, genital, and perianal areas) were observed in 58.82% (*n* = 10/17) of the individuals. No characteristic clinical signs were noted in the remaining 41.18% (*n* = 7/17). When available, skin samples (*n* = 9), whole blood (*n* = 6), feces (*n* = 1), and/or ocular swabs (*n* = 1) were used to confirm the viral infection.

### 2.2. Ectoparasite Identification

All ectoparasites were manually collected during comprehensive physical examinations performed by trained veterinarians immediately after the animals’ admission to the health center. Ticks and lice were carefully removed from the porcupines’ integument and promptly preserved in labeled sterile microtubes containing 70% ethanol for subsequent morphological and molecular analyses.

The ectoparasites were morphologically identified under a stereomicroscope using established taxonomic keys [24,25].

Ticks of the species *Amblyomma longirostre* were identified through distinct life-stage characteristics. Nymphs were recognized by their extensively shagreened (rugose) scutal surface with few large, deep lateral punctations and a sharply pointed hypostome [24]. Adult specimens exhibited a dark brown scutum with greenish and coppery patches, paired short spurs on coxa I (with the external spur approximately one-third the length of the segment and the internal spur half as long), and five ventral sclerotized areas near the festoons consisting of one median elongated area and four lateral areas [25]. Additional diagnostic features included a broadly hexagonal dorsal gnathosoma and hypostome with 3/3 dentition [25,26].

The species *Amblyomma sculptum* (formerly classified as *A. cajennense*) was differentiated by its lighter brown scutum with whitish or coppery markings, unequal spurs on coxa I, and a long hypostome with 3/3 dentition [25,27].

Lice were identified as belonging to the genus *Eutrichophilus* based on their elongated body morphology, host specificity to porcupines, and characteristic features of the antennae and legs [28,29].

While morphological identification remains reliable for *Amblyomma* nymphs and adult ticks, we adopted a conservative taxonomic approach for immature stages, identifying larvae only to genus level (*Amblyomma* spp.) unless specimens were reared to adulthood under controlled laboratory conditions, with species confirmation subsequently achieved through observation of definitive morphological characteristics in molted adults.

### 2.3. Molecular Methods

Prior to DNA extraction, all ectoparasites underwent three sequential washes with absolute ethanol, with three-minute intervals between washes, to minimize potential contamination from porcupine skin. Ectoparasites were pooled by host and taxonomic group, with ticks and lice processed separately. When multiple specimens of the same tick species were collected from a single host, they were grouped into species-specific pools. Pool sizes varied according to the number of ectoparasites collected per host, with detailed counts of ticks (including life stages) and lice per host provided in Table 1.

For sample preparation, ticks were transferred individually or in pools to sterile microtubes and flash-frozen in liquid nitrogen. Mechanical disruption was performed using sterile needles and pipette tips, followed by homogenate preparation in nuclease-free water (70:30 tick/water ratio). To ensure complete lysis, samples underwent three freeze-thaw cycles alternating between liquid nitrogen immersion and incubation at 55 °C in a thermoblock. After centrifugation at 12,000× *g* for 16 min at 15 °C, 200 µL of supernatant was subjected to DNA extraction using the Wizard™ Genomic DNA Purification Kit (Promega, Madison, WI, USA).

PCR amplification was performed using Platinum™ PCR SuperMix (Life Technologies, Carlsbad, CA, USA) with pan-pox primers targeting a conserved region of the putative metalloproteinase gene [30]. Reaction controls included DNA from a porcupine skin sample confirmed to be infected with BPoPV (GenBank accession no. MN692191) as the positive control and nuclease-free water as the negative control.

PCR products were purified with ExoSAP-IT™ (Applied Biosystems, Santa Clara, CA, USA) and sequenced bidirectionally on a Genetic Analyzer 3500 (Applied Biosystems) using BigDye™ Terminator v3.1 chemistry. Sequence quality was assessed using FinchTV 1.4.0, with subsequent editing and assembly performed in BioEdit v7.2.5 [31], employing the Cap-contig algorithm.

### 2.4. Phylogenetic Analysis

Alignments and nucleotide identities were obtained using BLAST^®^ 2.13.0 and Clustal W in BioEdit v7.2.5, respectively [31]. The Maximum Composite Likelihood phylogenetic tree was constructed using MEGA software v.11.0.10. The set of aligned nucleotide sequences was submitted to the Find Best-Fit Substitution Model in MEGA to determine the best evolutionary model [32].

### 2.5. Multiple Sequence Alignment

A multiple sequence alignment was performed using the MUSCLE algorithm implemented in UGENE v.45.1 to assess the genetic similarity among the viral sequences [33]. To investigate the potential functional implications of nucleotide variations, all sequences were translated in silico into amino acid sequences using BioEdit v.7.2.5 [25].

## 3. Results

**Figure 1:**
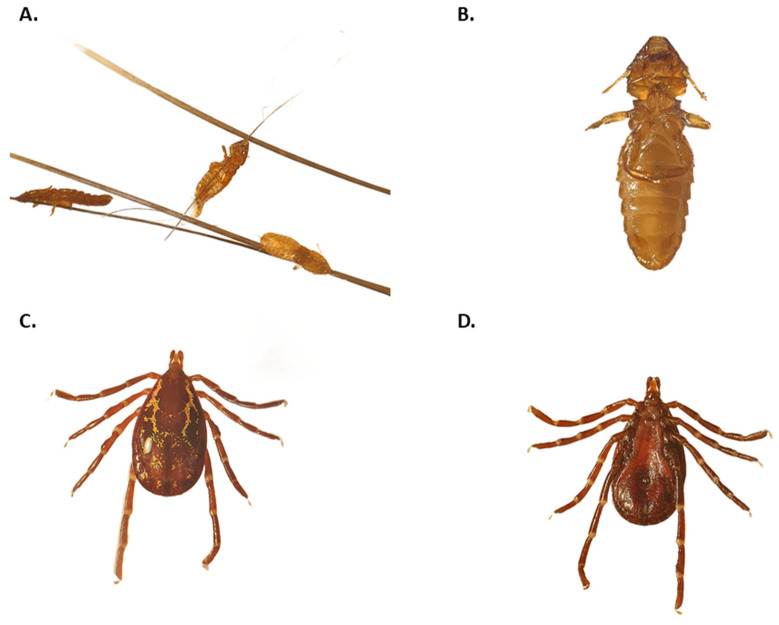
Morphological identification of ectoparasites collected from Brazilian porcupines (*Coendou* spp.). (**A**) Lice (*Eutrichophilus* sp.) attached to porcupine spines, visualized under a stereomicroscope. (**B**) A single chewing louse (*Eutrichophilus* sp.), showing a dorsoventrally flattened body and short antennae, consistent with characteristics of the genus. (**C**) Adult *Amblyomma longirostre* tick, showing a dark brown scutum with green and coppery spots, a hexagonal gnathosoma base, and short spurs on coxa I. (**D**) Adult *Amblyomma longirostre*, exhibiting a light brown scutum with whitish ornamentation and unequal spurs on coxa I.

As all the animals sampled were parasitized by several ticks and lice (Figure 1), a total of 84 ticks in various life stages were found, including adults of *Amblyomma longirostre* [*n* = 26] and *A. sculptum* [*n* = 2] ticks, nymphs of *A. longirostre* [*n* = 1] and *A. sculptum* [*n* = 13], and larvae of *Amblyomma* spp. [*n* = 41], as well as *A. longirostre* larvae [*n* = 1]. Additionally, lice of the genus *Eutrichophilus* were found on the *C. spinosus* from Santa Catarina State, totaling 10 lice. Ticks and lice were found attached to the spines of the porcupines, but none were located at the lesion sites.

**Figure 2:**
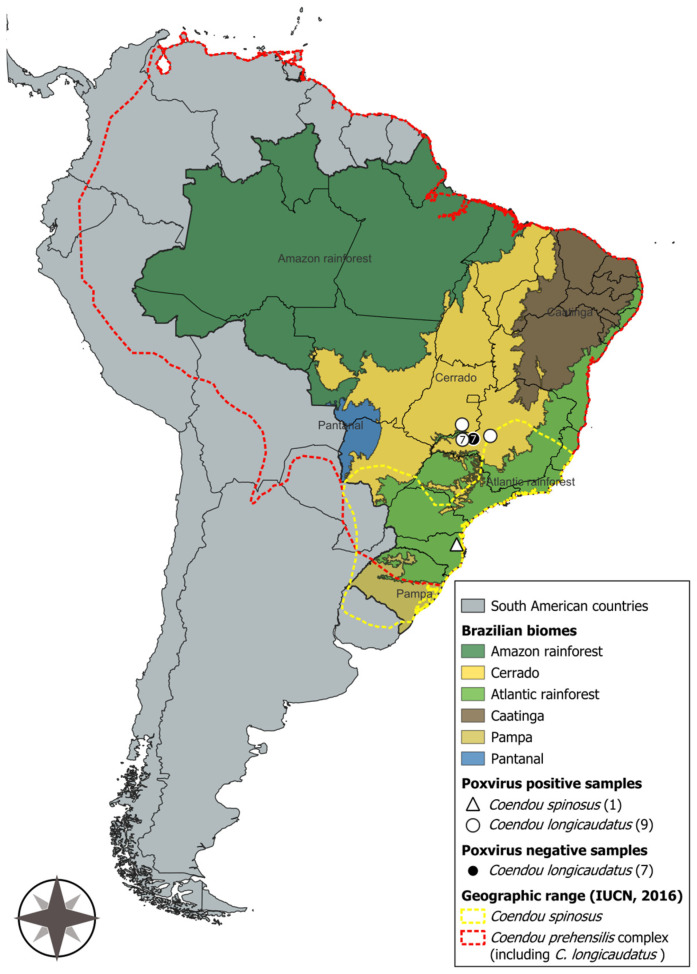
Map of South America highlighting Brazil, its biomes, and the geographic ranges of *Coendou spinosus* and the *Coendou prehensilis* complex, including *C. longicaudatus* [3,34]. Brazilian porcupinepox virus (BPoPV) samples are represented by white geometric shapes, while negative samples are indicated by black geometric shapes. Map created using QGIS software (version 3.44, “Solothurn”).

**Figure 3:**
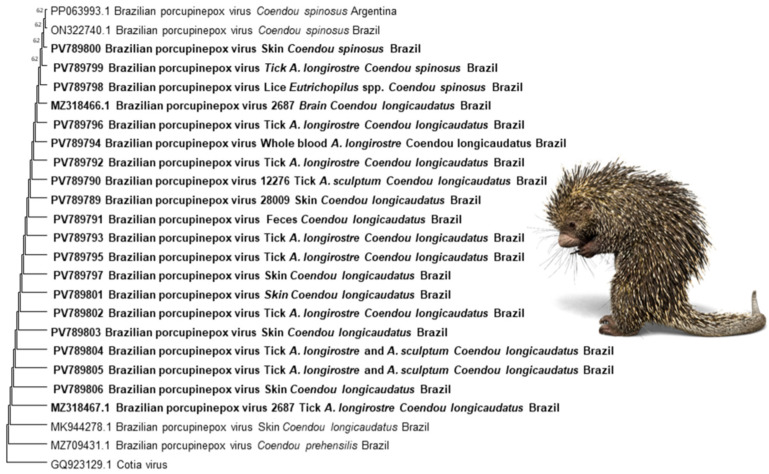
The evolutionary history was inferred using the Neighbor-Joining method. The optimal tree is shown. The percentage of replicate trees in which the associated taxa clustered together in the bootstrap test (1000 replicates) is shown next to the branches. The evolutionary distances were computed using the Maximum Composite Likelihood method and are expressed as units of the number of base substitutions per site. This analysis involved 25 nucleotide sequences. All ambiguous positions were removed for each sequence pair (pairwise deletion option). There was a total of 134 positions in the final dataset. Evolutionary analyses were conducted in MEGA11 [32]. GenBank accession numbers are shown next to each sequence. Sequences in bold were obtained in the present study. The image depicts the Brazilian porcupine (*Coendou* spp.), the host species, and was adapted using licensed elements from Canva Pro.

Among the porcupines presenting cutaneous lesions compatible with BPoPV infection (*n* = 10), 80% (*n* = 9/10) of skin samples or blood, 90% (*n* = 9/10) of pooled *Amblyomma* ticks, and 100% (*n* = 1/1) of pooled lice samples tested positive for poxvirus. A pool of *Amblyomma longirostre* ticks collected from an asymptomatic host that tested negative for BPoPV DNA yielded a positive result for BPoPV DNA. The resulting sequences exhibited 100% nucleotide identities with the BPoPV metalloproteinase gene strain reference (GenBank MN692191.1). Comprehensive data for each host is provided in Table 1, the geographic locations of the sampled animals are displayed in Figure 2, and the phylogenetic tree is shown in Figure 3.

**Figure 4:**
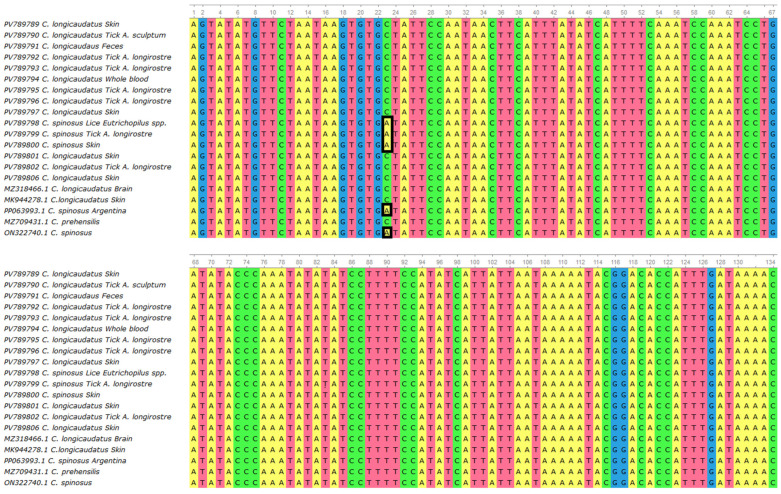
Multiple sequence alignment generated using MUSCLE (UGENE v.45.1) [27], including 20 BPoPV sequences from this study and four publicly available BPoPV sequences from GenBank. The alignment shows high nucleotide conservation among most sequences, except for a non-synonymous substitution at position 23 (highlighted), where sequences derived from *Coendou spinosus* exhibit a cytosine (C) in place of an adenine (A).

**Figure 5:**
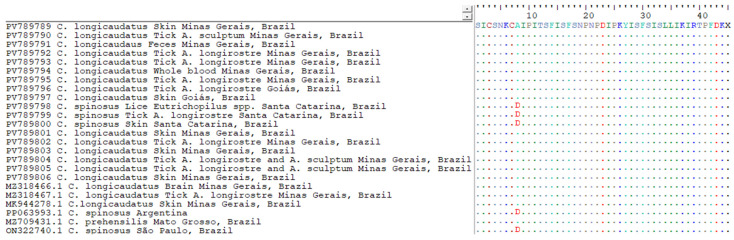
Amino acid alignment of the translated metalloproteinase gene fragment from BPoPV sequences, generated using BioEdit v.7.2.5 [25]. Reference sequence is shown in full, with identical residues in other sequences represented by dots. A non-synonymous substitution is highlighted (in red) at position 8, where sequences derived from *Coendou spinosus*—including those from Santa Catarina State (PV789800, PV789799, and PV789798), São Paulo State (PP063993), and Argentina (ON322740)—display an aspartic acid (D) residue, in contrast to alanine (A) in all other samples.

The multiple sequence alignment revealed a high level of conservation across the metalloproteinase gene fragment among all BPoPV sequences analyzed. However, a non-synonymous nucleotide substitution at position 23 was consistently identified in all sequences from *Coendou spinosus* and their ectoparasites (Figure 4). In these samples, a cytosine (C) was observed in place of the adenine (A) found in all other sequences from *C. longicaudatus* and its associated ectoparasites. This substitution results in a missense mutation, changing the predicted amino acid from alanine (A) to aspartic acid (D) (Figure 5).

## 4. Discussion

Our study provides the first molecular evidence of Brazilian porcupinepox virus (BPoPV) DNA in both clinically affected and asymptomatic *Coendou* spp. porcupines, as well as in their ectoparasites: *A. longirostre*, *A. sculptum* ticks, and *Eutrichophilus* spp. lice. The consistent detection of viral DNA across multiple host–parasite systems suggests these arthropods may serve as potential vectors in BPoPV transmission cycles. *A. longirostre* emerged as the predominant ectoparasite, with both tick species and lice testing positive for BPoPV regardless of the host’s clinical status. However, other routes of virus transmission or the involvement of other ectoparasites cannot yet be ruled out.

While *A. sculptum* is typically associated with terrestrial hosts [35], we identified this tick species in 40% (4/10) of clinically affected porcupines, whereas only *A. longirostre* was detected in asymptomatic individuals. This distribution pattern may reflect behavioral modifications in infected animals, as eyelid lesions and secondary bacterial conjunctivitis likely impair their arboreal capabilities, forcing prolonged ground contact. Interestingly, while we detected BPoPV DNA in *A. longirostre* across multiple hosts, adult *A. sculptum* ticks (reared from engorged nymphs collected from PCR-positive hosts) showed no evidence of viral DNA, suggesting inefficient transstadial transmission in this species.

Of particular note, we observed a discordant case in which an asymptomatic, PCR-negative porcupine harbored BPoPV-positive *A. longirostre* ticks. This host remained clinically normal for over 70 days and tested negative across all sampled tissues (blood, skin crusts, lesion swabs, feces), suggesting two possible scenarios: (1) the ticks acquired the infection from a previous host, supporting the potential for mechanical transmission; or (2) the porcupine may represent a resistant or transiently exposed individual capable of clearing the infection while maintaining ectoparasite BPoPV DNA positivity. This finding highlights the complex dynamics of BPoPV ecology, where subclinically infected hosts and their ectoparasites may contribute to silent viral maintenance.

The transmission dynamics of poxviruses by arthropod vectors are well documented in related systems. Mechanical (intrastadial) and transstadial transmission of lumpy skin disease virus (LSDV, genus *Capripoxvirus*) have been demonstrated in *Rhipicephalus decoloratus* and *A. hebraeum* ticks, with transovarial transmission reported for *R. appendiculatus* [19,20,36]. Phylogenetically, LSDV and BPoPV cluster within a major poxvirus clade based on whole-genome analyses [6], suggesting potential parallels in their transmission mechanisms.

Porcupines maintain a unique ecological relationship with ticks, particularly with *A. longirostre* and *A. parkeri*, which are frequently associated with Erethizontidae hosts [37,38,39]. While adult *A. longirostre* predominantly parasitize porcupines, its immature stages (larvae and nymphs) are primarily found on wild Passeriformes [37]. This bimodal host preference suggests porcupines may serve as both key hosts for adult tick maintenance and potential reservoirs supporting juvenile tick development [38]. The solitary nature of porcupines and their limited interspecific interactions make this tick–host system particularly intriguing. The parasitism of Passeriformes by juvenile *A. longirostre* may represent an understudied interspecific transmission route that could facilitate BPoPV spread across ecological niches. This hypothesis is further supported by the documented capacity of other poxviruses (e.g., LSDV) for both intrastadial and transstadial transmission in tick vectors.

The close ecological association between porcupines and human settlements raises significant concerns about potential zoonotic spillover pathways. This concern is amplified by multiple factors: the documented parasitism of zoo veterinarians by *Amblyomma* species originating from porcupines [40], the frequent occurrence of porcupines as roadkill throughout Brazil [41], and their regular presence in peri-urban environments. These animals are increasingly admitted to wildlife rehabilitation centers following human-related injuries, creating artificial but consequential interfaces with humans, domestic animals, and other wildlife species. While the zoonotic capacity of BPoPV remains uncharacterized, this epidemiological context—particularly the virus’s detection in both porcupines and their ectoparasites—suggests a plausible spillover risk that demands rigorous investigation.

The epidemiological parallels between swinepox virus (SWPV) and Brazilian porcupinepox virus (BPoPV) transmission are particularly noteworthy. Field observations have implicated the pig louse (*H. suis*) and stable flies (*Stomoxys calcitrans*) in swinepox virus (SWPV) outbreaks, with their role as potential mechanical vectors hypothesized in both Brazil and Italy [17,18]. These findings gain additional relevance from documented SWPV cases in wild boars (*Sus scrofa*) with heavy louse infestations in Italy, where fatal infections presented with characteristic poxvirus lesions [16]. The proposed mechanical transmission mechanism—where lice-induced pruritus facilitates viral entry through excoriated skin during host-to-host contact—may have direct relevance to BPoPV ecology. When considered alongside the solitary behavior of wild porcupines, these collective findings strongly support the involvement of ectoparasites in BPoPV transmission dynamics. The similar pathological presentations between SWPV and BPoPV infections, combined with the demonstrated vector capacity of lice in other poxvirus systems, suggest that *Eutrichophilus* lice and *Amblyomma* ticks may play analogous roles in maintaining and spreading this highly pathogenic poxvirus among porcupine populations.

Our sequence analyses identified a consistent non-synonymous mutation in the BPoPV metalloproteinase gene that appears specific to *Coendou spinosus* infections. This single-nucleotide polymorphism results in an alanine-to-aspartic acid (A → D) substitution at a conserved residue and was detected in all three study specimens from Santa Catarina State, Brazil, as well as in two additional *C. spinosus*-derived BPoPV sequences from São Paulo State (Brazil) and Argentina. The mutation’s exclusive presence in *C. spinosus* across this broad geographic range (spanning >1000 km) suggests either (i) a host-adapted viral variant circulating in this porcupine species or (ii) a distinct phylogeographic lineage with possible adaptive significance. Given that viral metalloproteinases are often involved in viral replication [42], this amino acid change—located within a functionally relevant domain—may influence virus–host interactions or pathogenic potential. Although further functional studies are needed to clarify its biological impact, this substitution may represent an early molecular signature of host adaptation or diversification in this emerging Neotropical poxvirus.

While our sample size of ectoparasites (84 ticks and 10 lice from 17 porcupines) may appear limited, this study represents the first systematic investigation of potential arthropod involvement in BPoPV transmission. The challenges of sampling these elusive, arboreal rodents and the rarity of confirmed BPoPV cases make this dataset particularly valuable. In fact, our collection effort exceeds sample sizes from comparable foundational studies of poxvirus transmission in domestic and wild hosts under natural conditions [16,17,18]. Thus, the data generated here provide valuable insights and establish a baseline for future studies aiming to experimentally confirm the vector competence of these ectoparasites in BPoPV transmission and to expand our understanding of its ecological and epidemiological features.

## 5. Conclusions

Our study provides the first molecular evidence of Brazilian porcupinepox virus (BPoPV) DNA in ectoparasites (*Amblyomma longirostre*, *A. sculptum* ticks, and *Eutrichophilus* sp. lice) collected from Neotropical porcupines (*Coendou longicaudatus* and *C. spinosus*). The frequent detection of viral DNA in ectoparasites from symptomatic hosts, coupled with the finding of BPoPV-positive ticks on an asymptomatic, PCR-negative porcupine, strongly suggests these arthropods may participate in viral transmission cycles. The detection of a consistent non-synonymous mutation (alanine-to-aspartic acid) in the metalloproteinase gene of all *C. spinosus*-derived BPoPV sequences suggests potential host-associated viral diversification. While these field observations suggest that *A. longirostre*, *A. sculptum*, and *Eutrichophilus* sp. may be potential vectors, experimental studies are now required to establish transmission mechanisms and to evaluate whether the observed genetic mutation influences viral tropism or transmission efficiency. These findings significantly advance our understanding of BPoPV ecology and highlight the complex interactions between this emerging poxvirus, its porcupine hosts, and their ectoparasites in Neotropical ecosystems.

## Figures and Tables

**Table 1:** Host identification, porcupine species, geographic location, presence of clinical signs consistent with BPoPV infection, identification and quantification of ectoparasites, PCR results for BPoPV, and GenBank accession numbers.

Id	Species	City, State	Clinical Signs	Ectoparasites	PCR	Genbank
2687	*Coendou longicaudatus*	Indianópolis, MG	Yes	*A. longirostre* (*n* = 1)	Skin + Tick +	MZ318467 MZ318466
10928	*C. longicaudatus*	Uberlândia, MG	Yes	*A. longirostre* nymphs (*n* = 1) *A. sculptum* adult (*n* = 1)	Skin + Tick +	PV789806 PV789805
12276	*C. longicaudatus*	Uberlândia, MG	Yes	*A. sculptum* (*n* = 1)	Skin + Feces + Tick +	PV789791 PV789790
13454	*C. longicaudatus*	Araguari, MG	Yes	*A. longirostre* male adult (*n* = 1) *A. sculptum* nymphs (*n* = 1)	Skin + Tick +	PV789803 PV789804
6754	*C. longicaudatus*	Patos de Minas, MG	Yes	*A. longirostre* male adult (*n* = 2)	Skin + Tick +	PV789801 PV789802
23502	*C. longicaudatus*	Caldas Novas, GO	Yes	*A. longirostre* male adult (*n* = 2) Larvae *Amblyoma* spp. (*n* = 38)	Skin + Tick +	PV789797 PV789796
24147	*C. longicaudatus*	Araguari, MG	Yes	*A. longirostre* male adult (*n* = 3)	Tick +	PV789795
24347	*C. longicaudatus*	Uberlândia, MG	Yes	*A. longirostre* male adult (*n* = 1)	Tick + Whole blood +	PV789793 PV789794
41412023	*Coendou spinosus*	Blumenau, SC	Yes	*A. longirostre* male adult (*n* = 2) *Eutrichopilus* spp. (*n* = 10)	Skin + Tick + Lice +	PV789800 PV789799 PV789798
28009	*C. longicaudatus*	Uberlândia, MG	Yes	*A. sculptum* nymphs * (*n* = 12)	Skin + Ticks −	PV789789
26284	*C. longicaudatus*	Uberlândia, MG	No	*A. longirostre* male adult (*n* = 2)	Tick + Whole blood − Ocular swab −	PV789792
27965	*C. longicaudatus*	Uberlândia, MG	No	*A. longirostre* male adult (*n* = 1)	Skin − Ticks −	-
30774	*C. longicaudatus*	Uberlândia, MG	No	*A. longirostre* female adult (*n* = 1) *A. longirostre* larvae (*n* = 1)	Tick − Whole blood −	-
33765	*C. longicaudatus*	Uberlândia, MG	No	*A. longirostre* male adult (*n* = 4)	Tick − Whole blood −	-
34481	*C. longicaudatus*	Uberlândia, MG	No	*A. longirostre* male adult (*n* = 4)	Tick − Whole blood −	-
34716	*C. longicaudatus*	Uberlândia, MG	No	*A. longirostre* (*n* = 2)	Tick −	-
38966	*C. longicaudatus*	Uberlândia, MG	No	Larvae *Amblyomma* spp. (*n* = 3)	Tick − Whole blood −	-

Caption: Id: Identification; GO, Goiás State; MG, Minas Gerais State; SC, Santa Catarina State. * The ticks were monitored through their molting from nymphs to adults over a period of 27 days, resulting in the identification of seven adult females and five adult males of *Amblyomma sculptum*. Subsequently, the adult ticks were tested and found to be negative.

## Data Availability

The nucleotide sequences generated during the study have been deposited in GenBank under the following accession numbers: MZ318466-MZ318467 and PV789789-PV789806. This study was registered in the Sistema Nacional de Gestão do Patrimônio Genético (SISGEN) under the number AAB53C6.
